# Differential Gut Microbiome Profiles in Long-Distance Endurance Cyclists and Runners

**DOI:** 10.3390/life14121703

**Published:** 2024-12-23

**Authors:** Guy Shalmon, Rawan Ibrahim, Ifat Israel-Elgali, Meitar Grad, Rani Shlayem, Guy Shapira, Noam Shomron, Ilan Youngster, Mickey Scheinowitz

**Affiliations:** 1 Sylvan Adams Sports Institute, School of Public Health, Tel Aviv University, Tel Aviv-Yafo 6997801, Israel; 2 Faculty of Medical and Health Sciences, Tel Aviv University, Tel Aviv-Yafo 6997801, Israel; 3 Department of Biomedical Engineering, Faculty of Engineering, Tel Aviv University, Tel Aviv-Yafo 6997801, Israel; 4 Edmond J. Safra Center for Bioinformatics, Tel Aviv University, Tel Aviv-Yafo 6997801, Israel; 5 Pediatric Infectious Diseases Unit, The Center for Microbiome Research, Shamir Medical Center, Tel Aviv-Yafo 6997801, Israel

**Keywords:** gut microbiome profile, gut microbiota, long-distance endurance athletes, cyclists, runners

## Abstract

We recently have shown that the gut microbiota composition in female and male runners positively correlates with sports, and female runners show similar gut microbiome diversity to male runners. However, gut microbiota composition has not yet been fully investigated in other endurance athletes, such as cyclists. Therefore, in the current study, we investigated the gut microbiome profiles in competitive, non-professional female and male cyclists compared to what we have shown in runners. We aim to understand (1) whether the gut microbiome signature is sport-specific; (2) whether there is a microbiome difference between female and male cyclists and runners; and (3) whether the gut bacteria expressed in cyclists and runners correlates with exercise performance. Our study included 58 subjects: 18 cyclists (9 males), 22 runners (13 males), and 18 control subjects (9 males). Fecal samples were obtained and subjected to taxonomic analysis to assess the relative abundances of species across subjects based on 16S rRNA sequencing results. Both alpha and beta diversity of the bacterial communities were evaluated to identify compositional variations between the groups. Each participant completed a maximal oxygen consumption test and a time-to-exhaustion test at 85% of the measured VO2max. Cyclists performed the test on an SRM ergometer, while runners used a motorized treadmill. Blood lactate levels were measured at 5 min intervals throughout the time-to-exhaustion trials. Alpha diversity demonstrated a significant difference (*p-adj* < 0.001) between cyclists and runners. Male cyclists showed significantly lower alpha diversity than runners (*p-adj* < 0.001). The taxonomic analysis of gut microbiota composition between cyclists, runners, and controls showed a lower or higher abundance of fifteen different bacteria. In cyclists, there was a significant positive correlation between six bacteria, and in runners, there was a significant positive correlation between eight bacteria, with weekly training volume, time-to-exhaustion, VO2max, and blood lactate levels. This study suggests potential sport-specific characteristics in long-distance cyclists’ and runners’ gut microbiome signatures. These findings emphasize the differences in gut microbiota between cyclists and runners, probably due to the difference in physiological and biomechanical conditions related to the activity mode during each sport.

## 1. Introduction

Recently, we have shown that the gut microbiota composition in competitive, non-professional female and male runners is positively correlated with sports performance [1]. These findings suggested that gut microbiota may be crucial in athletic performance, potentially influencing energy metabolism, inflammation, and recovery. In addition, studies have shown that the microbiome of athletes is characterized by a higher amount of short-chain fatty acids, which can be energy substrates during exercise [2].

In studies examining the relationship between exercise and the microbiome, most data have focused on runners, with considerably less attention given to other endurance athletes. Research on the microbiome of competitive cyclists remains limited. For instance, Petersen et al. [3] investigated the gut microbiota of both professional and amateur cyclists, revealing that the proportion of *Prevotella* bacteria in their microbiota increased with training intensity. These microorganisms metabolize carbohydrates and amino acids, including branched-chain amino acids [3,4]. Their pilot study offered the first insight into the gut microbiota of cyclists, uncovering significant correlations between the microbial taxa present in professional cyclists and those associated with high exercise intensity.

The study of the gut microbiome profiles in endurance athletes from various sports disciplines, including cyclists and runners, and the conditions during exercise that may influence gut bacterial composition represent an intriguing area of research. This is particularly relevant because different sports impose distinct biomechanical forces on the athlete, which may, in turn, impact the gut. For example, while runners engage in dynamic movements involving jumping and landing during exercise, cyclists maintain a seated position with a fixed body posture during their activity. However, the potential influence of gut microbiota on athletic performance underscores the need for further research. Specifically, it is better to characterize the microbiota of athletes across different sports disciplines and investigate how these microbial communities vary between sports that involve distinct physical demands.

Moreover, most gut microbiota studies are predominantly focused on male subjects. Research comparing the gut microbiome profiles of females and males in the general population is sparse and yields inconclusive results. However, human and animal studies have identified sex-based differences in the microbial composition [5,6,7,8]. Comparative studies between male and female athletes are even less common. Regarding the gastrointestinal microbiome, there appears to be a difference between females and males, which may be attributed to variations in estrogen levels [9]. In contrast, our previous study found no difference in the overall microbiome composition between female and male competitive runners [1]. This raises important questions regarding the differences in microbiome composition between male and female cyclists. Additionally, it is crucial to investigate whether there are distinctions in the microbiome profiles of male runners compared to cyclists and between female runners and cyclists.

The current study aimed to determine the gut microbiota composition in long-distance endurance female and male cyclists and runners, to define whether the gut microbiome signature is sport-specific, and to examine the correlation between gut bacteria in cyclists and runners and exercise performance indicators, such as VO2max, blood lactate levels, and time-to-exhaustion. Additionally, the study explored potential microbiome differences between female and male cyclists and runners.

## 2. Materials and Methods

### 2.1. Study Design and Participants

We recruited long-distance endurance cyclists and runners from competitive sports groups, while participants in the control group were recruited from the general population.

Fifty-eight subjects participated in the study (31 males and 27 females). They included 18 cyclists (9 males), with a mean age of 46 ± 7 years; 22 runners (13 males), with a mean age of 43 ± 6.5 years; and 18 control subjects (9 males), with a mean age of 41 ± 7.4 years. The cyclists and runners were amateur, competitive, non-professional athletes who competed at the domestic level, and their training was intense. The cyclists were defined as competitive endurance athletes who cycle at least 120 km per week, and the runners as competitive endurance athletes who run at least 50 km per week. The control group performed light physical activity with a weekly running volume of less than 5 km per week.

Each participant filled out an online survey detailing their weekly training regimen, including the number of training days per week, total duration per session, exercise intensity (expressed as a percentage of maximum heart rate), and eating habits (types of foods consumed, portion sizes, and meal frequency). This information was gathered to assess their dietary patterns, which may influence the composition of the gut microbiota. Based on this information, we included participants whose diet consisted of meat, fish, fruits, vegetables, and grains in more or less similar quantities throughout the week, without any dramatic deviations or significant differences in quantities between one participant and another. The ethnic origin of all the participants in this study was Ashkenazi Jews (originating from Europe and North America). Therefore, it can be assumed that no significant cultural differences among them would lead to substantial variations in the types of food consumed. To minimize variability in diet, only omnivorous participants were included. Individuals who had taken supplements, such as probiotics, prebiotics, multivitamins, antacids (e.g., beta-alanine, sodium bicarbonate, among others), or antibiotics within three months before the study were excluded.

Each subject received information about the study and signed an informed consent form after approval from the Tel Aviv University Ethics Committee, Israel (approval No. 0003766-1). All informed consent forms signed by the subjects are in the files of the principal researcher at Tel Aviv University, Israel.

### 2.2. Exercise Tests

Each participant performed a maximal exercise stress test to evaluate their aerobic fitness level. Maximal oxygen consumption (VO2max) was measured using a cycling test on a stationary ergometry (SRM) for cyclists or a running test on a treadmill (H/P Cosmos) for the runners and the control group, with gas exchange analyses using a COSMED Quark metabolic cart (COSMED S.r.l., Rome, Italy) [10,11].

The cyclists were instructed to begin cycling at a workload of 80 watts, maintaining an average pedaling cadence of 80 revolutions per minute. The workload was increased by 20 watts every minute, while the average cadence was kept constant throughout the exercise until exhaustion.

The runners and controls were instructed to begin running at a speed corresponding to 50% of their assessed running economy, and the speed progressively increased every minute until reaching exhaustion.

A week later, each subject performed a sub-maximal exercise test at 85% of the measured VO2max until exhaustion to determine ‘time-to-exhaustion’ [12]. The runners perform the test on the H/P Cosmos treadmill, and the cyclists on the SRM ergometer. Heart rate (HR) was monitored using a POLAR watch (Polar Electro Oy, Kempele, Finland). Capillary blood lactate levels were measured from fingertip sampled every 5 min during the time-to-exhaustion test using a Lactate Scout+ hand-held analyzer (EKF Diagnostics GmbH, Barleben, Germany).

### 2.3. Gut Microbiome Analysis

The detailed description of stool sample collection, DNA extraction, PCR protocol, sequencing, and analysis is described in Shalmon et al. [1].

Briefly, fecal samples were collected using sterile stomacher^®^ bags, aliquoted, and stored at −80 °C. DNA was extracted using the MagCore Genomic DNA Tissue Kit and amplified with custom primers targeting the V4 region of the 16S rRNA gene. Sequencing was performed on a MiSeq platform, and the resulting data were processed using the QIIME2 pipeline for quality filtering, OTU clustering, and diversity analyses.

### 2.4. Statistical Analyses

A comparison of cardiorespiratory measures, weekly training volume, and body mass index (BMI) among the three groups (cyclists, runners, and controls) was performed utilizing One-Way ANOVA in IBM SPSS Statistics (version 29), supplemented by post hoc Bonferroni analysis. Furthermore, sex differences were assessed through independent-sample *t*-tests, also conducted in IBM SPSS Statistics (version 29).

For the gut microbiome analysis, non-parametric statistical techniques were applied, assuming non-normal distribution and unequal variance. The Kruskal–Wallis test was used to compare the differences among groups (male vs. female, cyclists, runners, and controls). Alpha diversity was evaluated using the estimate_richness function and Faith’s Phylogenetic Diversity (Faith’s PD). For beta diversity, unweighted and weighted UniFrac distances and Bray–Curtis dissimilarities were computed, and principal coordinate analysis (PCoA) was employed to visualize these distances. Differential abundance in the gut microbiome was determined using DESeq2 (version 1.36.0) from the R/Bioconductor package (version 3.19). Taxa were assigned to the lowest possible taxonomic classification, with differential abundance assessed based on adjusted *p*-values < 0.05 and |log2FoldChange| ≥ 0.58. Sex was incorporated as a blocking factor in the differential analysis. Boxplots were generated using ggplot2 (version 3.4.4), while correlations with clinical parameters were computed using the corr.test function from the psych package (version 2.3.9) and visualized using the corrplot package (version 0.92).

Further details of the gut microbiome statistical analyses are provided in our previous publication [1].

## 3. Results

### 3.1. Participants’ Characteristics and Cardiopulmonary Exercise Tests

As expected, the cyclists’ and runners’ cardiopulmonary exercise test results were higher than the controls. A statistically significant difference was observed in the First Ventilatory Threshold (VT1), with the runners showing a significant difference from the controls (*p-adj* < 0.001) and cyclists (*p-adj* < 0.015). Similarly, for the Second Ventilatory Threshold (VT2), both the cyclists and runners exhibited significant differences compared to the controls (*p-adj* < 0.001). The VO2max was significantly higher in both the runners (*p-adj* < 0.001) and cyclists (*p-adj* = 0.007) compared to the controls. Finally, time-to-exhaustion was significantly higher in runners compared to controls (*p-adj* < 0.001), and cyclists showed a marginally significant difference (*p-adj* = 0.05) compared to controls. In addition to the cardiometabolic measures, blood lactate levels revealed a statistically significant difference only between the cyclists and the control group (*p-adj* = 0.018), with no significant difference found between the runners and controls (*p-adj* = 0.7). While there was a difference in the weekly training volume between the cyclists (174 ± 54 km) and runners (67 ± 15 km), no differences were found in the VT2 or VO2max. However, a significant difference was found in the VT1 (*p-adj* = 0.015) and time-to-exhaustion (*p-adj* = 0.042). Table 1 presents each experimental subject’s characteristics.

In our comparison of male and female cyclists and runners, we observed that male runners exhibited higher time-to-exhaustion than male cyclists (*p* = 0.03). Similarly, female runners had higher time-to-exhaustion than female cyclists (*p* = 0.05). However, when analyzing the VT1 and VO2max—two other critical metrics in exercise testing for assessing athletic performance—we found no statistically significant differences between female cyclists and female runners (VT1, *p* = 0.7; VO2max, *p* = 0.6) or between male cyclists and male runners (VT1, *p* = 0.3; VO2max, *p* = 0.8). The characteristics of the female and male runners and cyclists are presented in Table 2.

### 3.2. Microbiome Findings

**Figure 1:**
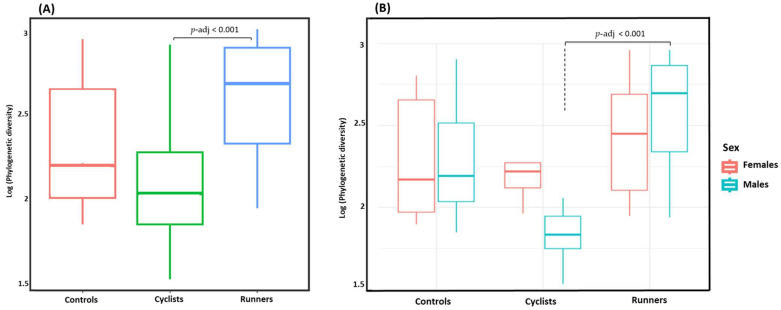
(**A**) Bacterial alpha diversity assessment between the cyclists, runners, and controls. While a difference was observed between the runners and the controls, the difference was more pronounced between the runners and the cyclists. The cyclists exhibited a lower species richness compared to the runners. (**B**) Comparison of the bacterial alpha diversity between sexes across the cyclists, runners, and controls. A statistically significant difference was observed only when the male cyclists were compared to the male runners.

Alpha diversity was significantly lower in cyclists than in runners (*p-adj* < 0.001). While alpha diversity was significantly higher in runners than controls (*p-adj* = 0.04), no difference was observed between cyclists and controls (*p-adj* = 0.18) (Figure 1A). A comparison of alpha diversity between females and males within each group did not reveal any statistically significant differences. However, male runners exhibited significantly higher alpha diversity than male cyclists (*p-adj* < 0.001) (Figure 1B).

Beta diversity is a key metric for characterizing the differences in microbial community composition between samples, reflecting the degree of dissimilarity in species distribution across ecological niches or experimental groups. We investigated beta diversity differences between cyclists and runners. Our findings revealed that Principal Coordinate Analysis (PCoA) based on unweighted UniFrac distance demonstrated distinct group clustering (*p-adj* = 0.002). However, no significant differences were observed in analyses using weighted UniFrac distance or Bray–Curtis dissimilarity (*p-adj* = 0.8 and *p-adj* = 0.3, respectively). Furthermore, no differences in beta diversity were found between females and males within any of the groups.

**Figure 2:**
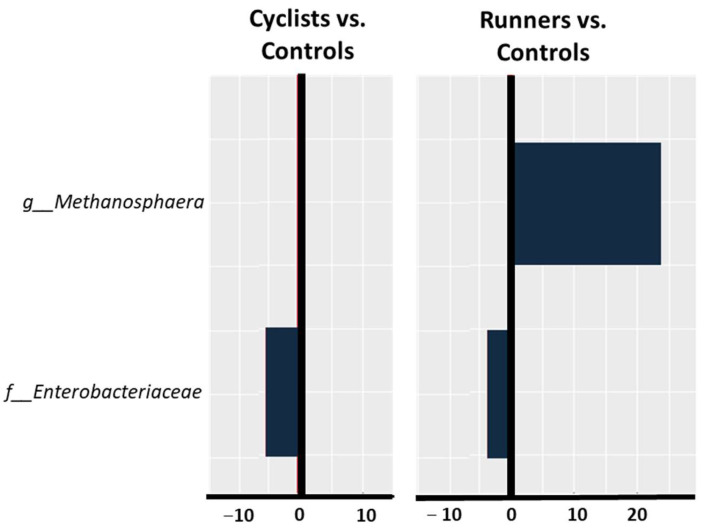
Taxonomic gut microbiota profiling in the cyclists compared to the controls and in the runners compared to the controls. The *X*-axis represents log2FoldChange, a commonly used metric in microbiome analysis to quantify the relative change in abundance of specific taxa between different conditions. A log2FoldChange greater than zero indicates an increased abundance, while a value less than zero indicates a decreased abundance.

Taxonomic gut microbiota profiling revealed distinct microbial patterns in cyclists and runners. Compared to the controls, the runners exhibited a significantly lower abundance of *Enterobacteriaceae* at the family level (log2FoldChange = −3.9, *p-adj* = 0.0001) and a significantly higher abundance of *Methanosphaera* at the genus level (log2FoldChange = 24.01, *p-adj* = 5.64 × 10^−20^). However, the cyclists demonstrated only a significantly lower abundance of *Enterobacteriaceae* (log2FoldChange = −5.42, *p-adj* = 2.29 × 10^−5^). The difference in gut microbiota composition between the cyclists and controls versus the runners and controls is shown in Figure 2.

The male cyclists, compared to the male controls, at the family level, demonstrated a significantly higher abundance of *Coriobacteriaceae* (log2FoldChange = 2.5, *p-adj* = 0.019) and a significantly lower abundance of *Enterobacteriaceae* (log2FoldChange = −6.7, *p-adj* = 9.39 × 10^−6^) and *Leuconostocaceae* (log2FoldChange = −7.7, *p-adj* = 0.05). At the genus level, the male cyclists demonstrated a significantly higher abundance of *Bifidobacterium* (log2FoldChange = 3.67, *p-adj* = 0.019) and *Pseudomonas* (log2FoldChange = 4.86, *p-adj* = 0.019), and a significantly lower abundance of *Catenibacterium* (log2FoldChange = −22.87, *p-adj* = 8.2 × 10^−13^). In a comparison between the male cyclists and male runners, at the genus level, the male runners exhibited a significantly higher abundance of *Catenibacterium* (log2FoldChange = 25.6, *p-adj* = 4 × 10^−19^) and *Methanosphaera* (log2FoldChange = 21.2, *p-adj* = 1.24 × 10^−11^).

**Figure 3:**
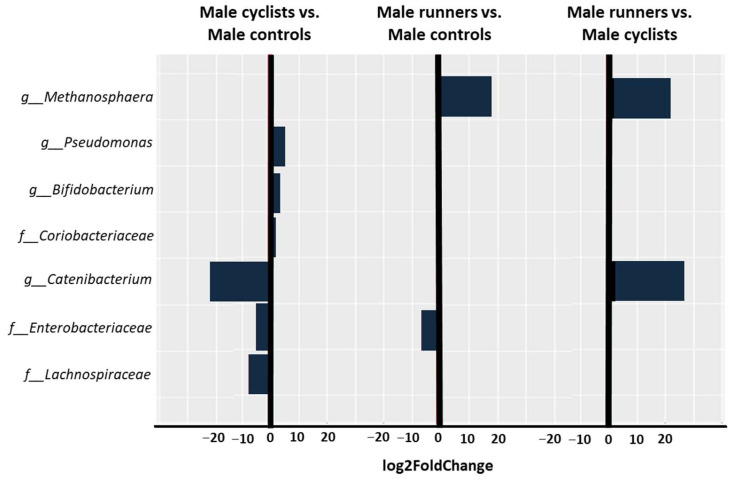
Taxonomic profiling of the gut microbiota at the family and genus levels was performed to compare the male cyclists vs. male controls, the male runners vs. male controls, and the male runners vs. male cyclists. A log2FoldChange less than zero indicates a decreased abundance, while a value greater than zero indicates an increased abundance.

The differences between the male cyclists, male runners, and male controls are shown in Figure 3.

The female cyclists, compared to the female controls, at the family level, demonstrated a significantly higher abundance of *Clostridiaceae* (log2FoldChange = 2.58, *p-adj* = 0.05), *Lachnospiraceae* (log2FoldChange = 1.12, *p-adj* = 0.05), and *Ruminococcaceae* (log2FoldChange = 1.6, *p-adj* = 0.05), as well as a significantly lower abundance of *Coriobacteriaceae* (log2FoldChange = −2, *p-adj* = 0.05) and *Gemellaceae* (log2FoldChange = −3.97, *p-adj* = 0.05). At the genus level, the female cyclists demonstrated a significantly higher abundance of *Dialister* (log2FoldChange = 5.26, *p-adj* = 0.05), *Mitsuokella* (log2FoldChange = 21.7, *p-adj* = 5.9 × 10−9), *Phascolarctobacterium* (log2FoldChange = 4.17, *p-adj* = 0.05), *Prevotella* (log2FoldChange = 3.86, *p-adj* = 0.05), and *Ruminococcus* (log2FoldChange = 1.09, *p-adj* = 0.05).

In a comparison between the female cyclists and female runners, at the family level, the female runners exhibited a significantly higher abundance of *Methanosphaera* (log2FoldChange = 36.5, *p-adj* = 2.28 × 10^−22^).

**Figure 4:**
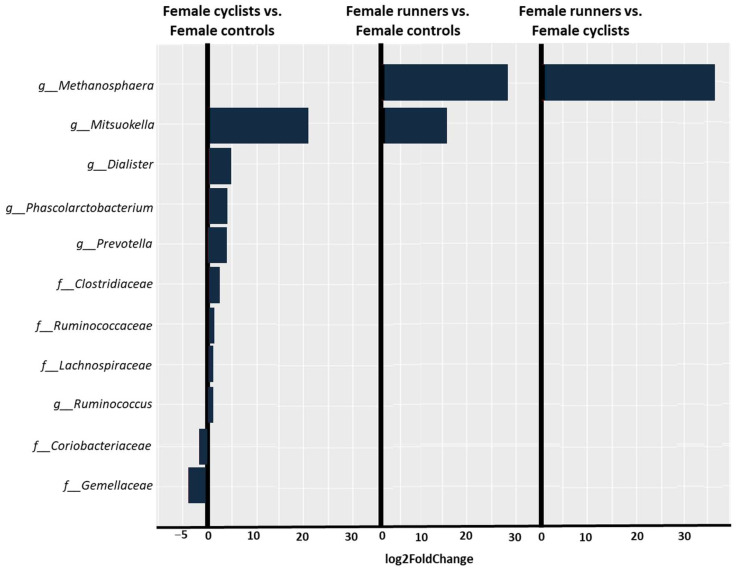
Taxonomic profiling of the gut microbiota at the family and genus levels was performed to compare the female cyclists vs. female controls, the female runners vs. female controls, and the female runners vs. female cyclists. A log2FoldChange less than zero indicates a decreased abundance, while a value greater than zero indicates an increased abundance.

The differences between the female cyclists, female runners, and female controls are shown in Figure 4.

The correlation between the microbiome composition and exercise performance is as follows. As mentioned above, the female cyclists and runners showed a significantly higher abundance of *Mitsuokella*, and the female cyclists showed a significantly higher abundance of *Dialister* and *Prevotella*. In this context, in cyclists, the presence of *Dialister* was positively correlated with lactate blood levels (r = 0.47, *p-adj* = 0.004) and time-to-exhaustion (r = 0.41, *p-adj* = 0.02), and the presence of *Prevotella* was positively correlated with weekly training volume (r = 0.5, *p-adj* = 0.03). In runners, the presence of *Mitsuokella* was positively correlated with VO2max (r = 0.41, *p-adj* = 0.05) and time-to-exhaustion (r = 0.41, *p-adj* = 0.03).

Table 3 presents (A) the top six positive correlations in the cyclists and (B) the top eight positive correlations in the runners between bacteria at the genus level and physiological measures in exercise. The table presents positive correlations (*p-adj* ≤ 0.05), exhibiting a linear association with a Pearson correlation coefficient greater than 0.4 (r > 0.4). It can be observed that the correlation results we obtained differ between the cyclists and the runners, with variations in the types of bacteria that correlated with their performance.

## 4. Discussion

In our laboratory, we investigate gut microbiota in endurance athletes and its association with sports performance under controlled laboratory conditions. In a previous study, we focused on runners. In the current study, we focus on cyclists and compare their results with those of runners, including a comparison between sexes.

Our recent study showed a positive correlation between gut microbiome composition and sports performance in female and male runners [1], with alpha diversity significantly higher in runners than in controls. The current study investigated whether gut microbiome is sport-specific among non-professional, competitive, long-distance endurance cyclists and runners. We hypothesize that the physiological and biomechanical conditions during running exercise and the absence of similar mechanical forces in cycling will lead to distinct gut microbiomes in these two groups. This study is essential as it provides insights into the potential influence of different sports on gut microbiota composition.

In this study, bacterial alpha diversity significantly differed between competitive, long-distance endurance cyclists and runners, with runners showing greater species richness than cyclists. No significant difference in alpha diversity was found between males and females in cyclists or runners, but male runners showed significantly higher alpha diversity than male cyclists. Alpha diversity is widely regarded as a key metric for evaluating gut microbiota health, with lower diversity associated with disease and adverse health outcomes [13]. This finding suggests that the unique stress conditions and mechanical forces during running exercise may increase gut microbiota bacterial strain richness and thus contribute to the runner’s performance, indicating a possible advantage for runners compared to cyclists.

Taxonomic gut microbiota profiling revealed a significantly higher abundance of *Methanosphaera* in runners but not cyclists. *Methanosphaera* is a non-motile archaeon known for its methane production [14]. Methane has been found to decrease intestinal transit rate [15]. A review by Triantafyllou et al. [16] found that methane production is linked to slower intestinal transit times. A positive correlation exists between breath methane positivity and delayed transit, regardless of health status.

We found that the abundance of *Catenibacterium* was higher in male runners and lower in male cyclists, which may explain differences in gastrointestinal symptoms, health effects, and exercise performance. The genus *Catenibacterium*, which includes the species *Catenibacterium mitsuokai*, is known for its production of short-chain fatty acids (SCFAs). The primary fermentation products of this bacterium are butyrate, iso-butyrate, lactic acid, and acetic acid [17]. Current research indicates that SCFAs could improve exercise performance by modulating muscle metabolism and glycogen storage. In animal studies, particularly in mice, SCFAs have been found to enhance endurance capacity during exercise [18]. SCFAs could increase muscle physical function by increasing muscle glycogen levels [19,20,21] and enhance muscle fibers’ metabolic efficiency by increasing ATP production [19,22].

Our study found time-to-exhaustion differences between male cyclists and male runners. The male runners had a higher TEE value, meaning they could sustain the effort for a longer time at an intensity of 85% of their VO2max (Table 2). This finding may be related to the fact that the abundance of *Catenibacterium* was higher in male runners than in male cyclists. Butyrate, the main product of *Catenibacterium*, is increasingly used as a supportive agent in preventing dysbiosis [22]. Yakovleva et al. demonstrated that oral supplementation with butyrate alleviated oxidative stress in the skeletal muscles of mice with dysbiosis, suggesting its potential therapeutic role in preventing muscle function deterioration [23].

Female cyclists showed a significantly higher abundance of *Prevotella*, *Lachnospiraceae*, *Phascolarctobacterium*, *Clostridiaceae*, *Dialister*, *Ruminococcaceae*, and *Ruminococcus*. Still, the expression of these bacteria was not observed among the female or male runners.

We found a positive correlation between *Prevotella* and weekly training volume in cyclists. Our finding is consistent with the results of Petersen et al. [3], who also found a significantly higher abundance of *Prevotella* in the gut microbiota of professional and amateur cyclists, and it was positively correlated with the weekly training volume of the competitive cyclists. In their study, cyclists training more than 11 h per week exhibited a greater relative abundance of *Prevotella* than those training fewer hours.

Female cyclists, not female or male runners, showed a significantly higher abundance of *Lachnospiraceae*. We also found a positive correlation between the genus *Lachnospira*, a member of the *Lachnospiraceae* family, and weekly training volume in cyclists. *Lachnospiraceae* are a family of obligately anaerobic bacteria that produce butyrate [24]. Butyrate, an SCFA, has attracted considerable interest for its pivotal role in supporting gut homeostasis. Various in vivo and in vitro studies have highlighted butyrate’s crucial role in regulating inflammation, immune reactions, and gut barrier integrity [25].

A significantly higher abundance of *Ruminococcaceae* was observed in female cyclists, whereas no such difference was found in either female or male runners. *Ruminococcaceae* is a butyrate producer and a bacteria involved in intestinal health [26]. Han et al. [27] also demonstrated that in 19 female athletes, comprising both elite and non-elite athletes, bacteria that produce SCFAs, such as *Ruminococcaceae*, were predominant in the microbiota of elite female athletes. A comprehensive review conducted by Dorelli et al. [28] investigated the potential influence of physical activity on the composition of the human gut microbiome, independent of dietary factors. The analysis included ten studies: five focused on athletes, three on physically active individuals, and two on sedentary participants participating in exercise programs. Most of these studies found that physically active individuals, particularly athletes, exhibited greater diversity and a higher abundance of *Ruminococcaceae* than their inactive counterparts. This genus is associated with various gut structural, protective, and metabolic roles [28].

Only female cyclists demonstrated a significantly higher abundance of *Phascolarctobacterium*, with no difference observed in female or male runners. These bacteria can synthesize SCFAs, such as propionate and acetate, and may be linked to the host’s emotional state [29].

Male cyclists, not male or female runners, showed a significantly higher abundance of *Bifidobacterium*. *Bifidobacterium* produces lactic acid and SCFAs (acetate, propionate, and butyrate) [30]. The microbial fermentation of carbohydrates in the gut leads to the production of lactic acid, which can then be utilized by specific bacteria, such as *Eubacterium* halli, *Veillonella atypica*, and *Anaerostipes caccae*, to generate acetyl-CoA. This intermediate is further metabolized to form butyrate, accompanied by the production of ATP [31]. Additionally, *Bifidobacteria*, through the breakdown of polysaccharides, can release compounds that foster butyrate synthesis, independent of lactate-consuming bacteria’s ability to utilize lactate [32] directly. Consequently, an increase in lactate-producing bacteria could promote greater availability of metabolic substrates for butyrate synthesis, potentially boosting ATP production and reducing fatigue. While the enhanced production of ATP through lactate utilization may theoretically support energy needs during exercise, it is more likely that much of this ATP is employed in maintaining intestinal homeostasis, limiting its direct impact on muscle function. An alternative mechanism suggests that a higher prevalence of lactate-producing bacteria in the presence of sufficient fiber intake may lead to elevated levels of microbial lactate. This lactate could then be transported across the gut epithelium into the bloodstream, which skeletal muscle can take up, fueling exercise efforts [33]. By enhancing the systemic availability of lactate, microbiome modulation could optimize energy utilization and potentially improve endurance performance. As part of the evidence to support our hypothesis that the gut microbiota in athletes is sport-specific, we found positive correlations between eight bacteria and physiological measures in exercise that appeared only in runners and six bacteria only in cyclists (Table 3). Among the runners, more bacteria correlated with VO2max, lactate blood levels, and time-to-exhaustion. Among the cyclists, most of the bacteria we found were mainly associated with weekly training volume.

Our research highlights the microbiome differences between competitive but not professional long-distance endurance female and male cyclists and runners. Future research should further elucidate the functional roles of the specific bacterial taxa identified in our study, particularly those associated with endurance performance. In particular, a deeper understanding of how the gut microbiome differs between runners and cyclists could offer insights into the sport-specific adaptations of the microbiota and their impact on performance. For example, the distinct biomechanical demands of running and cycling may shape the microbial communities in ways that influence energy metabolism, muscle recovery, and fatigue resistance. Investigating the mechanistic pathways through which these microbiome differences impact exercise capacity and recovery could open the door to novel strategies for optimizing athletic performance through targeted dietary or microbiome-modulating interventions tailored to each sport.

## 5. Limitations

Although this study provides important insights into the relationship between physical activity and gut microbiome composition, several limitations should be considered. While our study included participants with similar dietary habits and who did not consume dietary supplements in the three months before the study, we did not have strict control over their eating habits or specific diets, as diet plays a crucial role in shaping the gut microbiome composition. Furthermore, reliance on self-reported dietary data and weekly training volume may introduce recall or reporting biases. Additionally, the study’s cross-sectional design precludes the ability to assess causality between physical activity and microbiome composition. While we performed a thorough microbiome analysis, this study did not account for other factors, such as sleep, stress, and genetic predisposition, which may also influence gut microbiota. We also acknowledge the methodological limitations of our analysis. We chose to use Faith’s PD method to account for the phylogenetic relatedness of the community members and DESeq2 for differential abundance analysis of the gut microbiome. However, we recognize that other analytical methods, with distinct advantages, were not included in the current manuscript. Future longitudinal studies with more diverse populations and additional control over confounding factors will be valuable in addressing these limitations.

## 6. Conclusions

Our study emphasizes the differences in gut microbiome profiles between long-distance endurance cyclists and runners, possibly due to the difference in physiological and biomechanical conditions related to the activity mode during each sport. While these findings suggest potential sport-specific characteristics in the gut microbiota, further research is needed to fully understand the complex interactions between exercise modality and microbial composition to validate this claim and strengthen the hypothesis that the gut microbiome of endurance athletes may be sport-specific.

## Figures and Tables

**Table 1:** Characteristics of the participants.

	Cyclists (*n* = 18)	Runners (*n* = 22)	Controls (*n* = 18)
Sex			
Females	9 (50.0%)	9 (40.9%)	9 (50.0%)
Males	9 (50.0%)	13 (59.1%)	9 (50.0%)
Age			
Mean (SD)	45 (5.12)	43.3 (7.56)	39.4 (5.6)
BMI			
Mean (SD)	22.9 (3.25)	23.2 (2.61)	23.9 (4.01)
Weekly training volume (km)			
Mean (SD)	174 (54)	67 (15.6)	5 (0)
Cardiopulmonary indices			
VT1 (mL/kg/min)	31.36 ± 5.9	35.86 ± 4.4	26.47 ± 3.9
VT2 (mL/kg/min)	40.6 ± 9	43.2 ± 5.7	31.7 ± 4.5
VO2max (mL/kg/min)	44.62 ± 9.6	46 ± 6.7	36.7 ± 5.4
Time-to-exhaustion (min)	11.3 ± 3.9	15.43 ± 6.7	7.4 ± 3.1
Lactate max (mmol/L)	9.5 ± 2.6	8 ± 1.6	7.2 ± 2.8

**Table 2:** Characteristics of the female and male cyclists and runners.

	Female Cyclists (*n* = 9)	Male Cyclists (*n* = 9)	Female Runners (*n* = 9)	Male Runners (*n* = 13)
BMI				
Mean (SD)	21.49 (3.04)	24.22 (2.9)	21.06 (1.46)	24.61 (2.2)
Weekly training volume (km)				
Mean (SD)	170.55 (53.41)	177.77 (57.61)	61.11 (12.6)	71.15 (16.6)
Cardiopulmonary indices				
VT1 (mL/kg/min)	31.22 ± 5.99	31.51 ± 6.19	34.25 ± 4.37	36.97 ± 4.38
VT2 (mL/kg/min)	39.35 ± 8.02	41.86 ± 10.27	40.52 ± 4.33	45.06 ± 5.93
VO2max (mL/kg/min)	42.44 ± 8.3	46.8 ± 10.7	43.87 ± 5.4	47.5 ± 7.4
Time-to-exhaustion (min)	10.38 ± 4.11	12.21 ± 3.67	15.20 ± 7.83	15.59 ± 6.22
Lactate max (mmol/L)	8.45 ± 2.97	10.53 ± 1.91	7.71 ± 1.52	8.33 ± 1.79

**Table 3:** Physiological bacteria that are significantly correlated with exercise performance among cyclists and runners.

**Correlations**								
	**Vo2max (mL/kg/min)**		**Time-to-Exhaustion (min)**		**Blood Lactate Levels (mmol/L)**		**Weekly Training Volume (km)**	
	**r**	***p-adj***	**r**	***p-adj***	**r**	***p-adj***	**r**	***p-adj***
**A. Bacteria in Cyclists**								
*g_Dialister*			0.41	0.02	0.47	0.004		
*g_Prevotella*							0.5	0.03
*g_Sutterella*							0.54	0.01
*g_Butyricicoccus*							0.49	0.03
*g_Lachnobacterium*							0.48	0.04
*g_Lachnospira*							0.53	0.02
**B. Bacteria in Runners**								
*g_Mitsuokella*	0.41	0.05	0.41	0.03				
*g_Methanosphaera*			0.41	0.007	0.51	0.05		
*g_Megamonas*	0.45	0.03					0.43	0.04
*g_Prevotellaceae*	0.63	0.01					0.41	0.05
*g_Rothia*	0.65	0.002					0.41	0.05
*g_Bacteroides*					0.46	0.02		
*g_Oscillospira*			0.43	0.02				
*g_Odoribacter*					0.41	0.05		

## Data Availability

The data described in this article can be freely and openly accessed at Mendeley Data: https://data.mendeley.com/datasets/dnybvvshz2/1 (accessed on 19 December 2024).
